# Commentary: Will dexamethasone ever have a role in the management of chronic subdural hematomas?

**DOI:** 10.1002/brb3.3446

**Published:** 2024-02-26

**Authors:** Adam James Wells

**Affiliations:** ^1^ Department of Neurosurgery Royal Adelaide Hospital Adelaide Australia

**Keywords:** chronic subdural hematoma, dexamethasone, steroid

1

Based on the results of two multicenter randomized controlled trials published over the last few years in the *New England Journal of Medicine*, dexamethasone in the management of chronic subdural hematomas (CSDH) has now been shown to be associated with worse clinical outcomes, and its use is, therefore, seemingly contraindicated. But is it too early to state that steroids have no role in the management of CSDH? In the Dex‐CSDH trial (Hutchinson et al., 2020), the authors demonstrated that dexamethasone resulted in fewer favorable outcomes and more adverse events in patients being managed for CSDH compared with placebo, and last year the DECSA collaborators (Miah et al., 2023) published the results of their trial that was discontinued early due to safety concerns relating to the inability to demonstrate noninferiority of dexamethasone therapy compared with burr hole drainage. The pathophysiology of CSDH remains unclear; however, trauma with an associated chronic inflammatory component seems likely, in which case inflammation suppression is still at least theoretically plausible as a surgical alternative or adjunct to minimize hematoma recurrence and improve clinical outcomes. The well‐recognized adverse effects of dexamethasone however, coupled with the inappropriate application of nonoperative management for what should clearly be a surgical problem in the DECSA study (Markwalder grade ≥2; patients with hemiparesis), may have potentially tarnished the role of steroids in this increasingly important global neurosurgical problem.

Formal guidelines for the management of CSDH are lacking, and there is still very limited level 1 evidence available from randomized clinical trials to support best practices (Mehta et al., 2018). Holl's survey on national current practices on CSDH management in the Netherlands (Holl et al., 2022) published in *Brain and Behavior* in 2022 aimed to determine clinical practice attitudes in order to direct the establishment and implementation of practice guidelines and demonstrated that experiences among neurologists and neurosurgeons towards dexamethasone use as a primary therapy or surgical adjunct were mostly positive. Although the study was performed before the results of either the Dex‐CSDH or DECSA trials were available, they found that 98% of respondents would implement dexamethasone as standard of care (when appropriate) if steroids were ultimately shown by the DECSA investigators to be as effective as burr hole craniostomy with an associated 20% cost saving, or if dexamethasone was shown to be as effective as surgery and with both trial arms having a similar side effect profile. Given that the majority of Dutch neurosurgeons reported mostly positive personal experiences with dexamethasone as a primary or adjunct therapy and few negative experiences, it would be interesting to see how the DECSA results would change attitudes and prescribing practices within the same population. The results of the Holl study should not be surprising and are consistent with the findings of other clinical trials investigating perioperative dexamethasone use for the surgical management of CSDH. A prospective study by Cine published last year compared surgical outcomes in patients receiving dexamethasone therapy with those that did not and found no additional adverse effects with adjuvant steroid use as well as demonstrating a statistically significant reduction in hematoma recurrence by the second postoperative week (Cine, 2023). Most patients in both arms of the Cine study had a Markwalder grade of ≤2 with a median age of 73 years; therefore, when used as an adjunct rather than as a surgical alternative dexamethasone may be beneficial by minimizing future surgical episodes related to symptomatic hematoma recurrence and with no increase in associated adverse events in a subgroup of patients.

CSDH is a heterogeneous condition with a wide‐ranging patient presentation (Kolias et al., 2014), and different management options including both surgical and nonsurgical strategies remain appropriate depending on the individual circumstances of each patient. As such, blanket recommendations are rarely helpful; what is appropriate for one patient will certainly not be appropriate for all, and this increases the difficulty of implementing evidence‐based management guidelines. Although the current literature would suggest that dexamethasone therapy is not beneficial and may even be harmful in certain CSDH patients, it might be premature to suggest that steroids do not have a role in carefully selected subpopulations. Similar scenarios have been seen previously in the neurosurgical literature. The STICH investigators (Mendelow et al., 2013) demonstrated that medical management for intracerebral hemorrhage was superior to surgical evacuation prior to further subgroup studies showing that surgery for superficial lobar hemorrhages has a small but significant survival advantage. Similarly, the findings of the ARUBA investigators were ultimately criticized for being misleading on the superiority of medical management of unruptured cerebral arteriovenous malformations, in which microsurgery was subsequently shown to improve longer term outcomes in carefully selected patients (Feghali & Huang, 2020). Although dexamethasone may at present have a questionable role in treating CSDH based on the current evidence, critical interpretation of the results of these recent trials raises the possibility that field advancement is being inappropriately delayed by diverting attention away from inflammation suppression in CSDH management. The DECSA authors should be encouraged to analyze and present their data relating to the Markwalder Grade 0 or 1 patients, the logical subgroup to detect noninferiority when compared with surgery. Nonsurgical management of CSDH will probably become an increasingly important topic of discussion over the coming years in conjunction with an aging population, and various drugs will no doubt be proposed; dexamethasone as an anti‐inflammatory may not ultimately prove to be the best drug for this purpose, but it may still be too soon to consign it completely to history.

## AUTHOR CONTRIBUTIONS

**Adam James Wells**: Conceptualization; investigation; writing—original draft; writing—review and editing.

## CONFLICT OF INTEREST STATEMENT

Adam Wells is the Abbie Simpson Clinical Fellow of the Neurosurgical Research Foundation; at no time has Adam Wells received payment or support in kind for any aspect of the submitted work.

### PEER REVIEW

The peer review history for this article is available at https://publons.com/publon/10.1002/brb3.3446.

## Data Availability

Data sharing is not applicable to this article as no datasets were generated or analyzed during the current study.
